# The Breathomics Profile of Volatile Sulfur Compounds in the Bipolar Spectrum, Does It Represent a Potential Tool for Early Diagnosis?

**DOI:** 10.3390/jcm14062025

**Published:** 2025-03-17

**Authors:** Federica Sancassiani, Mauro Giovanni Carta, Diego Primavera, Massimo Tusconi, Antonio Urban, Laura Atzori, Caterina Ferreli, Elisa Cantone, Gloria Virginia Cuccu, Goce Kalcev, Germano Orrù, Flavio Cabitza, Serdar M. Dursun, Cesar Ivan Aviles Gonzalez, Pedro José Fragoso Castilla, Shellsyn Giraldo Jaramillo, Giulia Cossu, Alessandra Scano

**Affiliations:** 1 Department of Medical Sciences and Public Health, University of Cagliari, Monserrato Blocco I (CA), 09042 Cagliari, Italy; federicasancassiani@yahoo.it (F.S.); maurogcarta@gmail.com (M.G.C.); diego.primavera@unica.it (D.P.); a.urban@aoucagliari.it (A.U.); elisa.cantone@libero.it (E.C.); glovirgicuccu@gmail.com (G.V.C.); gocekalcev@yahoo.com (G.K.); giuliaci@hotmail.com (G.C.); 2 PhD Program in Tropical Medicine, Universidad Popular del Cesar, Valledupar 200001, Colombia; pedrofragozo@unicesar.edu.co; 3 Department of Nursing, Universidad Popular del Cesar, Valledupar 200001, Colombia; 4 University Hospital of Cagliari, 09042 Cagliari, Italy; 5 Dermatology Clinic, Department of Medical Sciences and Public Health, University of Cagliari, 09042 Cagliari, Italy; atzoril@unica.it (L.A.); ferreli@unica.it (C.F.); 6 Department of Surgical Sciences, University of Cagliari, Cittadella Universitaria, 09042 Cagliari, Italy; alessandrascano@libero.it; 7 Fondazione per la Tutela dell’Identità Ogliastrina e della Barbagia di Seulo, Corso Vittorio Emanuele II, Perdasdefogu, 08046 Nuoro, Italy; cabitza.flavio@gmail.com; 8 Neurochemical Research Unit, Department of Psychiatry, University of Alberta, Edmonton, AB T6G 2G5, Canada; dursun@ualberta.ca; 9 Department of Medicine and Surgery, University of Kore, Piazza dell’Università, 94100 Enna, Italy; cesar.aviles@unikore.it; 10 Microbiology Program, Universidad Popular del Cesar, Valledupar 200001, Colombia; 11 Faculty of Health, Universidad Popular del Cesar, Valledupar 200001, Colombia; shellsyngiraldo@unicesar.edu.co

**Keywords:** bipolar disorders, BD, laboratory medicine, applied biomedical technologies, bipolar spectrum, prevention, new technologies, hyperactive, early diagnosis

## Abstract

**Background/Objectives:** Emerging laboratory technologies, such as breathomics, may enhance the early diagnosis of psychiatric disorders, including Bipolar Disorder (BD). This study investigates the detection of volatile sulfur compounds (VSCs) in exhaled breath as potential biomarkers for BD, comparing VSC levels between individuals with BD, healthy controls, and individuals with non-pathological hyperactivity. **Methods:** A matched case–control study was conducted involving 24 patients with BD and 95 healthy controls recruited at the University Hospital of Cagliari. Controls were selected using a matched-pair design based on age (±5 years) and sex through a block-matching technique to ensure comparability with cases. Participants underwent psychiatric interviews, completed the Mood Disorder Questionnaire (MDQ), and had their exhaled breaths analyzed for VSCs using a gas chromatograph (OralChroma™). Controls were selected and randomized for age and sex. **Results:** Patients with BD exhibited significantly higher levels of methyl mercaptan (CH_3_SH) compared to healthy controls (18.62 ± 5.04 vs. 9.45 ± 18.64 ppb, *p* = 0.022). Among individuals without BD, those with positive MDQ scores showed lower levels of CH_3_SH than those with negative scores (9.17 ± 5.42 vs. 15.05 ± 18.03); however, this difference did not reach statistical significance (*p* = 0.254), highlighting how the deep connection between some clinical and laboratory aspects needs to be investigated more thoroughly. **Conclusions:** The results suggest a correlation between oral dysbiosis and metabolic alterations in patients with BD, with CH_3_SH levels being higher in cases compared to controls. Further studies are needed to validate the use of VSCs as potential biomarkers for BD and to investigate their role in individuals with non-pathological hyperactivity.

## 1. Introduction

In the 1970s, carbon disulfide poisoning was a crucial field of occupational medicine [1,2]. The presence of methyl sulfide in exhaled air can indicate carbon disulfide poisoning [3], but adequate techniques for measuring it in exhaled air were not yet widespread. Those full-scale studies therefore used the method of detecting metabolites in tissues and blood [4].

A study from those years found a close association between the presence of carbon disulfide metabolites and excess suicide [5], but at the time, no one advanced the hypothesis that carbon disulfide intoxication could be a co-factor in people with traits or pathologies highly predisposing to suicide such as bipolar spectrum disorders. Indeed, according to occupational medicine studies, workers exposed to carbon disulfide were at risk of developing psychiatric symptoms, including acute psychotic episodes (with mania), rapid mood changes, uncontrollable anger, and irritability [6,7]. Based on current knowledge, these symptoms would draw attention to the bipolar spectrum [8,9,10]. Nevertheless, in the 1970s and 1980s, the spectrum of Bipolar Disorders was considered very narrow in the United States compared to European psychiatry [11]; the presence of psychotic symptoms therefore brought attention to the possible relationship between sulfate metabolites in the air and schizophrenia. Studies have therefore focused on schizophrenia [12,13]. A study found that the gradients of pentane in alveolar and the carbon sulfide in expired air were higher in a group of people diagnosed with schizophrenia than in the control groups [12]. One of the control groups consisted of people with psychiatric diagnoses other than schizophrenia and showed intermediate rates between people with schizophrenia and people without a psychiatric diagnosis. However, these were very small samples that did not allow for inferences to be made about diagnoses other than schizophrenia. The authors concluded that “Schizophrenia may be accompanied by accelerated lipid peroxidation in cell membranes” [12]. It should be emphasized that lipid peroxidation induced by oxidative stress has been found as an element in the pathophysiology of Bipolar Disorders (BPDs) and that mood-stabilizing drugs are known to counteract lipid peroxidation [14,15].

More recent investigations have suggested that the link between exposure to carbon disulfide and psychiatric disorders may extend beyond schizophrenia [16], with increasing evidence implicating oxidative stress and lipid peroxidation in the pathophysiology of Bipolar Disorder as well [17]. Oxidative stress is known to contribute to neuronal dysfunction, cellular damage, and altered neurotransmission, all of which are key components of mood dysregulation in Bipolar Disorder [18]. Furthermore, exposure to environmental toxins such as carbon disulfide can exacerbate underlying vulnerabilities in individuals predisposed to affective disorders, potentially acting as a catalyst for the onset of manic or depressive episodes [19].

Furthermore, metabolic dysregulation, another area of emerging interest in Bipolar Disorder research, has been linked to oxidative stress-related damage. Studies have shown that patients with Bipolar Disorder often present metabolic abnormalities [20,21,22], including elevated markers of inflammation and mitochondrial dysfunction, which can be exacerbated by environmental exposures [23]. In particular, the disruption of redox homeostasis and the generation of reactive oxygen species (ROS) could contribute to both the neuroprogression and the recurrent nature of Bipolar Disorder [24,25]. Since mood stabilizers, such as lithium and valproate, have been found to possess antioxidant properties and reduce lipid peroxidation [14,26], further exploration of the relationship between volatile sulfur compounds, oxidative stress, and Bipolar Disorder could offer new insights into the mechanisms of the disease and the potential development of biomarkers [27].

These findings emphasize the need for a broader reevaluation of historical studies that have primarily associated carbon disulfide exposure with schizophrenia, neglecting its potential impact on bipolar spectrum disorders [28]. Future research should focus on larger, well-characterized cohorts to determine whether individuals with Bipolar Disorder show distinct breathomic profiles indicative of increased oxidative stress or metabolic dysfunction [29,30,31]. The identification of specific volatile compounds associated with mood dysregulation could pave the way for innovative diagnostic approaches and contribute to a deeper understanding of the biological basis of Bipolar Disorder [32,33].

Recent research has noted that a series of volatile sulfur compounds (VSCs) are evaluable in the breath, and they are derived as metabolites from oral microbiota. This cavity contains bacterial reservoirs, such as the dorsum of the tongue, where anaerobic bacteria break down sulfur-containing amino acids like cysteine and methionine into volatile sulfur compounds (VSCs), such as hydrogen sulfide (H_2_S) and methyl mercaptan (CH_3_SH). It is thought that tongue coating is the primary source of VSCs. A microbiota imbalance in the oral cavity, where anaerobic opportunistic bacteria outnumber the commensal bacterial population, directly correlates with increased VSCs in the breath. This condition is called oral dysbiosis, and the primary clinical manifestation in these individuals is halitosis, or bad breath [34]. Furthermore, some unlinked researchers reported a close association between psychological conditions and the symptoms of patients complaining of halitosis [35,36]. These authors concluded that patients claiming to have halitosis may be experiencing excessive anxiety or be more vulnerable to neurosis because the condition is not always evident and cannot be interpreted as a real symptom.

Given these premises, it could be interesting to verify, thanks to modern techniques, whether different levels of sulfur compounds can be found in the air exhaled by people who have been diagnosed with Bipolar Disorder compared to people without a diagnosis of Bipolar Disorder [37,38,39,40].

Considering the ease of application of new technologies, it may also be interesting to evaluate, in the context of people who have no diagnosis of Bipolar Disorder, the levels of sulfur compounds in the air exhaled by people with non-pathological hyperactivity [41,42,43]. It has been hypothesized that three levels of hyperactivation can be recognized, identified by screening tools such as the Mood Disorder Questionnaire, with levels of adaptivity being higher than normal in the first level, adaptivity “in suffering” but still without a diagnosis of Bipolar Disorder in the second level, and with hyperactivity being associated with Bipolar Disorder only in the third level, respectively [41,42,43].

## 2. Methods

Design: Our study follows a matched case–control design. The cases were individuals diagnosed with Bipolar Disorder according to the DSM-5 criteria [44], while the controls were selected from individuals without a diagnosis of Bipolar Disorder [44]. The control group was created using block randomization matched for sex and age (±5 years) to ensure comparability between cases and controls in order to compare the level of carbon sulfide in the breaths of the two groups; another analysis was carried out by considering people without a diagnosis of Bipolar Disorder but positivity at Mood Disorder Questionnaire as cases and people without a diagnosis of Bipolar Disorder and negatives at the Mood Disorder Questionnaire as controls.

Sample and Recruitment: Over a three-day period, people attending the psychiatry and psychosomatic consultation center (for case selection) and the dermatology clinic (for control selection) of the Cagliari University Hospital were recruited and evaluated. After being given a detailed explanation of the research and signing the informed consent form, the subjects underwent a psychiatric interview and a general anamnesis and filled out the MDQ. The control group was selected through block randomization matching, ensuring that each case was paired with two controls of the same sex and within ±5 years of age. A control-matching cell was generated for each case, including all eligible individuals without a diagnosis of Bipolar Disorder. Two controls were randomly selected for each case within these matched cells. Once a control was selected, they were excluded from further eligibility to maintain independence between matching sets.

The inclusion criterion was patients aged between 20 and 69 years without any exclusion by sex.

The exclusion criteria were the inability to sign the informed consent form adequately and for the control group to be affected by Bipolar Disorder at the clinical interview. The control group of people without Bipolar Disorder was chosen after randomization by blocks based on the sample of cases. A matched control cell was created for each case with all the people without Bipolar Disorder of the same age (±5 years) and the sex of the overall sample of controls. Two controls were randomly selected for each cell. Once a block was placed on a subject without Bipolar Disorder, they were excluded from eligibility for the remaining blocks.

Study tools: The Mood Disorder Questionnaire (MDQ) is designed in principle to detect Bipolar Disorder [45]. More recently, however, after the instrument had proven to be poorly accurate, it was discovered that it could identify people with characteristics of the so-called bipolar spectrum [46], that is, people who fall into a range from individuals with characteristics of adaptive hyperactivity to people under stress but without a precise psychiatric diagnosis and people with frank Bipolar Disorder. In this case, the instrument is used to identify people without Bipolar Disorder, those who present traits of non-pathological hyperactivity [47].

The MDQ consists of a series of answers focusing on characteristics of behavior, mood, and thought commonly believed to be typical of Bipolar Disorder. A frequently used scoring threshold to determine whether someone has characteristics of the bipolar spectrum is to answer positively to seven or more out of the thirteen items on the tool [47,48]. If the questionnaire is used as a screener for Bipolar Disorders, having a score higher than this threshold suggests a likelihood of having Bipolar Disorder and the need for further clinical evaluation carried out by a professional.

Laboratory analysis of VSCs: In this study, we used patients’ morning breath as standard samples to detect volatile sulfur compounds [49], and therefore, we took VSC measurements for these patients between 9 and 11 a.m. Before the exam, the patients were instructed to avoid eating or drinking coffee, forgo their customary oral hygiene routine for the day of the measurements, and avoid consuming alcohol 12 h before the measurements. The VSC profiles and concentrations were determined using Gas Chromatograph OralChroma™ (Abimedical, Abilit Corp., Osaka, Japan) following the manufacturer’s instructions [50]. The breath samples were obtained using a disposable 1 mL syringe placed on the upper tongue of the patient. The subject was instructed to close their lips, and 1 mL of air was sampled. An amount of 1 mL of sample was injected into the measuring device, and after 8 min, the profiles of the three sulfur gases (H_2_S, CH_3_SH, and (CH_3_)_2_S) were displayed in part per billion (ppb), representing one microgram of VSC per liter of breath air (µg/L).

The first two are strictly related to oral microbiome metabolism, while the last is associated with systemic (i.e., gastric) illnesses [51].

Statistical Analysis: Data were collected anonymously using an I.D. number for each person recruited. A dedicated database was created. Given the matched case–control design, we used appropriate statistical methods for paired data. For continuous variables, we applied the paired *t*-test when the normality assumption was met, verified the results using the Shapiro–Wilk test, and used the Wilcoxon signed-rank test otherwise. For categorical variables, we chose to replace the Chi-square test with the McNemar’s test, which accounts for the dependence between paired observations. All statistical analyses were performed using Stata version 17 (StataCorp, College Station, TX, USA) [52].

Ethical Aspects: Institutional Review Board Statement (IRB) approval was not required for this study because the data were de-identified and made available to the public. This study was conducted according to the guidelines of the 1964 Helsinki Declaration. All study participants signed a written informed consent form after receiving a detailed description of the study (aims, procedures, and data protection), and they were aware of the possibility of the study being terminated at any time.

## 3. Results

The normality of continuous variables was assessed using the Shapiro–Wilk test, which indicated a non-normal distribution for all variables (*p* < 0.05). Consequently, we applied the Wilcoxon signed-rank test. The Wilcoxon test showed statistically significant differences between cases and controls for age (*p* = 0.0001) and CH_3_SH (*p* = 0.022). For categorical data, the McNemar test was used to account for the paired nature of the observations.

Table 1 shows the characteristics by sex and age of the selected sample. As can be seen, the large sample selected for the controls is older (67.75 ± 15.31 vs. 54.68 ± 15.79 years old; *p* < 0.0001) and has a lower frequency of women (36.8% vs. 62.5%; *p* = 0.022).

Table 2 shows the characteristics by sex and age and the level of CH_3_SH in breath air in the cases and matched controls. In this case, the matching technique made the samples homogeneous by sex and made the differences by age not statistically significant.

The comparison of age between cases and matched controls was performed using the Wilcoxon signed-rank test given that the normality assumption was not met (Shapiro–Wilk test; *p* < 0.05). The results revealed a statistically significant difference, with cases having a lower mean age (W = 1.00; *p* = 0.0001). Conversely, the analysis of CH_3_SH levels using the Wilcoxon signed-rank test showed a statistically significant difference between cases and controls (W = 9.00; *p* = 0.022). The distribution of sex (female) between cases and controls was analyzed using McNemar’s test, which showed no significant difference (χ^2^ = 0.00; *p* = 1.0000). These findings confirm the presence of significant age differences between cases and controls, while CH_3_SH levels and sex distribution remained comparable across groups.

Table 3 compares the level of CH_3_SH in breath air in people without Bipolar Disorder subdivided by MDQ positivity. People with MDQ+ show a lower level of CH_3_SH in breath air (9.17 ± 5.42 vs. 15.05 ± 18.03). However, the difference does not reach statistical significance (*p* = 0.254). The two samples are sufficiently homogeneous in terms of age, but among people with MDQ+, women are less frequent (12.5% vs. 40.5%). However, this difference does not reach statistical significance. The comparison between subjects classified as MDQ-positive (MDQ+) and MDQ-negative (MDQ−) was performed after verifying assumptions of normality (Shapiro–Wilk test). For age, both groups met the assumption of normality; therefore, a paired *t*-test was performed. No significant difference was found between groups (t = 0.078; *p* = 0.638). For CH_3_SH levels, a Wilcoxon signed-rank test was conducted due to the non-normal distribution of data. This analysis also did not indicate a statistically significant difference (W(15) = 0.05; *p* = 0.254). The distribution of females between the MDQ+ and MDQ- groups was compared using the McNemar test, revealing no statistically significant difference (χ^2^ = 0.05; *p* = 0.8159). These results suggest comparable demographic and biochemical characteristics between MDQ-positive and MDQ-negative groups.

## 4. Discussion

Our work has highlighted that a sample of people diagnosed with Bipolar Disorder has higher levels of CH_3_SH in their breath air than a control group without Bipolar Disorder matched for sex and age (Table 2). In the control group, people with hyperactivity episodes [53,54] (MDQ positive) but without a diagnosis of Bipolar Disorder did not show any differences compared to controls without hyperactivity episodes (MDQ negative), but on the contrary, they showed lower levels with a difference bordering on statistical significance (Table 3). This result is not discordant with the hypothesis that hyperactivity may have three different levels ranging from normal to pathological, of which only one is associated with the diagnosis of Bipolar Disorder [55,56,57]. In comparison, the two subthreshold levels would represent a level of adaptive hyperactivity and a level of hyperactivity linked to the dysregulation of rhythms and stress, therefore being considered maladaptive but not yet pathological in diagnostic terms [58,59,60].

Our studies of Bipolar Disorder are consistent with the findings from studies of people with schizophrenia over 30 years ago [12]. Given the long time that has passed and the changes in diagnostic systems, it remains to be determined whether this homogeneity is due to the broadening of the concept of Bipolar Disorder and the concomitant narrowing of the diagnosis of schizophrenia (which might suggest that it may be the same type of diagnosis in modified systems) or whether excess volatile sulfate compounds are common to schizophrenia and Bipolar Disorder [61,62,63,64].

The origin of endogenous CH_3_SH has yet to be discovered with certainty. A potential source could be the large intestine [65,66]. In this location, the metabolism of microorganisms, increasing in particular conditions, can produce volatile compounds containing sulfur. One of the conditions in which this occurs is advanced liver cirrhosis, as is known. The breathing of people suffering from this condition is characterized by the so-called “foetor hepaticus”, determined by high concentrations of such volatile organic compounds containing sulfur, among which methyl mercaptan and dimethyl sulfide are relevant [67,68].

Previous studies hypothesized that excess volatile sulfate compounds in the breath could be caused by the metabolism of certain neuroleptic drugs [12] or by a dysbiotic condition inside the oral microbiome. However, thioridazine and its derivatives, sulfide-containing neuroleptics, are no longer used practically and do not indicate Bipolar Disorder. Furthermore, the same ancient study denied an effect of neuroleptics because the use of these drugs was also widespread in the control sample with “other psychiatric diagnoses” [12]. Our study is a preliminary study conducted on a small sample; therefore, it has numerous areas for improvement. However, the data that have been highlighted deserve attention and suggest future investigations.

A first consideration concerns the alteration in metabolism that could be one of the causes of the excess of volatile sulfides in the breath and that, in Bipolar Disorder, seems to be related to the dysregulation of biological and social rhythms, which seems to be an intrinsic characteristic of the disorder [69,70]; this condition could also be responsible for a bacterial disbalance in the oral cavity due to, for example, a change in alimentary habits.

This hypothesis warrants further investigation because it may help identify a subset of individuals with hyper energy or hyperactivity who exhibit maladaptive rhythm dysregulation without a Bipolar Disorder diagnosis [71,72,73]. If a test on the emission of sulfides with the breath could identify this “at-risk” subpopulation, this could be a very useful biomarker [74,75,76].

However, at present, this is only a heuristic hypothesis that suggests the usefulness of future studies.

A second aspect worthy of being verified in the future is the liver conditions of people with Bipolar Disorder. It is known that Bipolar Disorder is associated with medical conditions that can compromise liver function [77]; excessive alcohol consumption [78] and some stabilizing drugs, such as valproate, can have unwanted effects on the liver [79,80].

Considering these limitations, the hypothesis remains that the typical accelerated lipid peroxidation in cell membranes in Bipolar Disorder may cause an excess of volatile sulfates and, thanks to new technologies, pave the way for the identification of early biomarkers.

## Figures and Tables

**Table 1:** Characteristics by sex and age of selected sample.

	Cases (N = 24)	Overall Controls (N = 95)	Statistics
Age	54.68 ± 15.79	67.75 ± 15.31	Wilcoxon signed-rank test W(23) = 1.00 *p* = 0.0001
Sex (female)	15 (62.5%)	35 (36.8%)	Df1, McNemar’s test χ^2^ = 5.145 *p* = 0.022

**Table 2:** Characteristics by sex, age, and level of CH_3_SH in breath air in cases and matched controls.

	Cases (N = 24)	Matched Controls (N = 48)	Statistics
Age (Mean ± SD)	56.08 ± 13.06	69.56 ± 11.05	Wilcoxon signed-rank test W(23) = 1.00 *p* = 0.0001
Sex (female) (Matching)	15 (62.5%)	30 (62.5%)	McNemar’s test Matching
CH_3_SH in breath air (Mean ± SD)	18.62 ± 5.04 ppb	9.45 ± 18.64 ppb	Wilcoxon signed-rank test W(23) = 2.00 *p* = 0.022

**Table 3:** A comparison of the level of CH_3_SH in breath air from people without Bipolar Disorder subdivided by MDQ positivity (with verification of the comparison by age and sex in the two groups).

	MDQ+ (N = 16)	MDQ− (N = 79)	Total Mean (N = 95)
Age	68.22 ± 17.80	57.92 ± 14.64	Paired *t*-test t = 0,078 *p* = 0.638
CH_3_SH in breath air	9.17 ± 5.42 ppb	15.05 ± 18.03 ppb	Wilcoxon signed-rank test W(15) = 0.05 *p* = 0.254

## Data Availability

The data presented in this study are available upon request from the corresponding author. Due to privacy and ethical issues, the data are not publicly available.
